# Values of heart rate at rest in children and adults living at different altitudes in the Andes

**DOI:** 10.1371/journal.pone.0213014

**Published:** 2019-02-28

**Authors:** Christian R. Mejia, Matlin M. Cárdenas, Dayanne Benites-Gamboa, Armando Miñan-Tapia, Gloria S. Torres-Riveros, Michael Paz, Yomayra Perez, José Rojas-Camayo

**Affiliations:** 1 Escuela de Medicina Humana, Universidad Continental, Huancayo, Perú; 2 Asociación Médica de Investigación y Servicios en Salud, Lima, Perú; 3 Facultad de Medicina, Universidad Ricardo Palma, Lima, Perú; 4 Universidad Privada de Tacna, Tacna, Perú; 5 Lincoln Medical and Mental Health Center, Bronx, New York, United States of America; 6 Instituto de Investigaciones de la altura, Universidad Peruana Cayetano Heredia, Lima, Perú; Nagoya University, JAPAN

## Abstract

**Introduction:**

The heart rate (HR) is useful for the monitoring of patients, but almost no studies have been found which describe their variations according to different geographic locales and altitudes using centiles in children and adults.

**Methodology:**

Descriptive, cross-sectional study of secondary data. Measurements were taken with a calibrated pulse oximeter; our participants resided in host cities for more than 2 months and underwent clinical evaluations by physicians. The results were categorized according to their age group and the altitude of residence using centile charts.

**Results:**

Our sample size consisted of 6,289 subjects across different villages in Peru. Using Pearson correlation between HR and altitude, it was found in the group of patients aged 1–5 years, a coefficient of -0.118 (p value = 0.012), in the group of patients aged 6–17, 0.047 (p value = 0.025), in the group of patients aged 18–50, -0.044 (p value = 0.041) and for the group of patients aged 51–80, 0.042 (p value = 0.256). In the groups of 1–5, 6–17 and 18–50 years of age, the variations were negligible but statistically significant due to our large sample size. When all of the data was evaluated, HR values were also found to have negligible variations according to the residence altitude, with a Pearson coefficient of -0.033 (p value = 0.009). Centiles charts were used to describe the distribution of HR for different age groups by altitude of residence.

**Conclusion:**

There are minimal variations of the HR according to the altitude of residence in all age groups.

## Introduction

A human being’s heart rate (HR) is one of the vital signs that is used in many clinical scenarios, like in anesthesiology, for example, where HR is one of the minimum monitoring data needed, reducing the risk of incidents by providing an early warning to reduce the risk of adverse events [1–3]. However, this vital sign may have variations according to multiple factors, one of the least studied being the variation due to the altitude where people reside. In the literature, there are almost no studies which evaluated its variability in patients residing at higher altitudes [4–7].

The objective of our study was to describe the heart rate values at rest in adults and children living at different altitudes in the Peruvian Andes and documenting it using centile charts.

## Methods

### Study design

An analytical cross-sectional study was performed using a secondary data analysis from a study that evaluated oxygen saturation at different altitudes in Peru, from sea level to the highest city in the world, located at an altitude of 5100 meters above sea level (masl) [8].

### Location and time

The selected sites were located in the central coast (Lima at 154 meters), south coast (Tacna at 562 meters), the southern Andes (Arequipa at 2335 meters and La Rinconada at 5100 meters), northern Andes (Yungay at 2500 meters), in the central jungle (Villa Rica at 1400 meters). The other 9 altitudes were located in the central Andes of Peru (San Jeronimo de Surco at 2000 meters, Paucartambo at 2880 meters, Huancayo at 3250 meters, Pachacayo at 3600 meters, San Pedro at 3950 meters, Junín at 4100 meters, Cerro de Pasco at 4338 meters, Huaron to 4500 meters and San Cristobal at 4715 meters).

### Population

Our patient population included ages from 1 to 80 years, who lived in the place of study for at least 2 months. Residents who had a medical history of chronic disease, chronic mountain sickness, cardiopulmonary disease, pregnancy, transfusion or donation of blood in the last 6 months were excluded. In addition, those with active respiratory or cardiac disease, and with physical and/or artificial impediment in the nail (malformation or enamel) where the heart rate reading could not be performed were also excluded.

### Data collection

The main variable was obtained using a pulse oximeter Nellcor (model N560). This device was used for obtaining all HR readings which were subsequently interpreted by a physician. To obtain the measurements, the participant or caregiver was first informed of the investigation and asked to participate. Once consent was obtained and the patient was accepted, they were asked to stay at rest for a few minutes. The OXI-P / I pediatric sensor was used for children and the DS-100A sensor for adults. Once HR was taken at rest, the participant underwent cardio-respiratory evaluation, performed by the same physician. If this evaluation did not find any evident abnormalities, the participant was classified as apparently healthy. After all this was performed, 6 measurements were taken with an interval of 10 seconds between each reading, to obtain an average heart rate for each participant.

### Statistical analysis

All of our variables were exported to the Microsoft Excel program for Windows 2010. Stata software version 11.1 was then used for the statistical analysis. In the descriptive statistics, the quantitative variables were represented by measures of central tendency (mean) and measures of dispersion (standard deviation); according to each altitude. For correlation analysis, statistics were obtained through the Pearson correlation (used to determine the relationship between heart rate and altitude in all the groups). For creating the percentiles, we used the following age groups: 1–5 years, 6–17 years, 18–50 years and 51–80 years.

### Ethics

The study was approved by an Institutional review board (IRB) committee and endorsed by the National Institute of Health of Peru (Committee of Ethics of the “Hospital Nacional Docente Madre Niño”, Office No. 0757-OADI-UI -HONODOMANI-SB-2015). At all times, ethics was respected for the investigation, the anonymity of the subjects that participated were kept confidential. The verbal consent of each adult subject was obtained and in the case of minors, we obtained consent from their parents.

## Results

Data from a total of 6289 people was analyzed. The average HR in all altitudes in children of 1–5 years old was 101.9 beats / min (n = 984), in children aged 6–17 years, it was 86.7 beats / min (n = 2363), in adults of 18–50, it was 76.2 beats / min (n = 2194) and in adults 51–80, it was 73.7 beats / min (n = 720). The mean resting heart rate ranged from 83.2 bpm (at 154m) to 90.4 bpm (at 5100 m). **(cf. Table 1)**

**Table 1:** Heart rate values by altitude and age range, in inhabitants at different altitudes.

Meters above sea level	1 to 5 years		6 to 17 years		18 to 50 years		51 to 80 years		Total	
	N	*HR	N	HR	N	HR	N	HR	N	HR
154	64	100,9	281	86,9	310	77,8	54	73,5	709	83,2
562	90	113,2	144	85,9	120	73,2	50	70,1	404	86,2
1400	68	98,7	146	90,8	28	72,9	72	74,9	314	87,3
2000	26	102,5	116	82,1	40	78,0	27	75,6	209	83,0
2335	70	100,5	171	87,0	239	77,6	42	74,6	522	83,5
2500	87	99,7	234	83,7	70	74,3	25	69,4	416	84,6
2880	95	102,5	122	86,0	134	79,3	53	74,2	404	86,1
3250	140	103,3	181	81,7	56	76,9	45	72,8	422	87,3
3600	30	90,8	117	83,1	136	75,9	78	72,8	361	78,8
3950	38	102,3	87	94,2	44	75,3	57	75,5	226	87,2
4100	91	100,5	116	91,9	297	77,8	138	76,1	642	83,2
4338	47	98,0	126	86,6	247	72,2	37	68,5	457	78,5
4500	36	97,4	121	86,2	353	75,1	15	73,3	525	79,1
4715	28	96,2	133	91,1	68	78,3	22	72,6	251	86,6
5100	74	105,4	168	88,0	52	78,4	5	73,8	299	90,4
**TOTAL**	984	101,9	2363	86,7	2194	76,2	720	73,7	6289	

*HR: Heart rate

**Fig 1:**
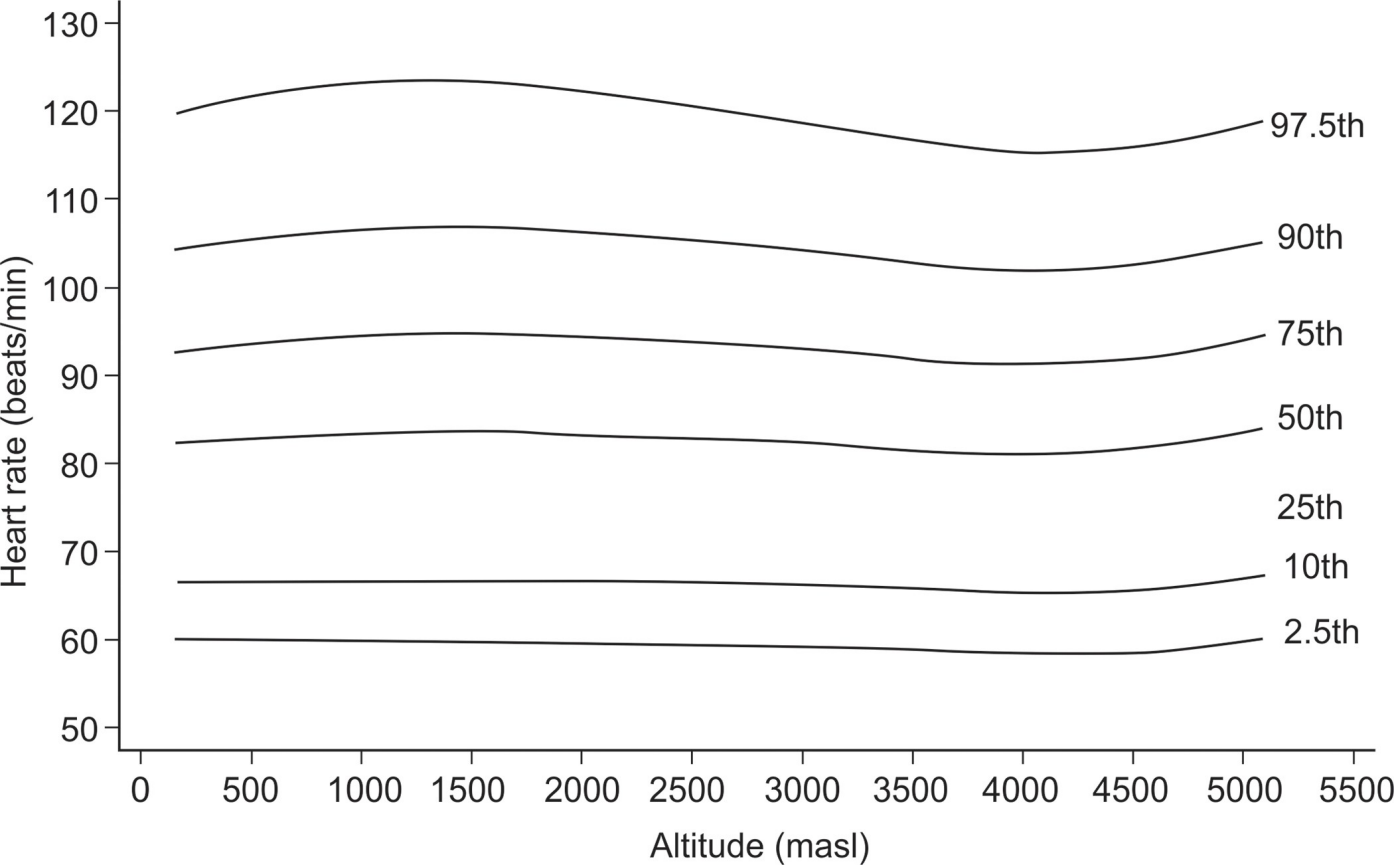
Heart rate at rest according to altitude of residence in Peruvian children of 1–5 years of age.

**Fig 2:**
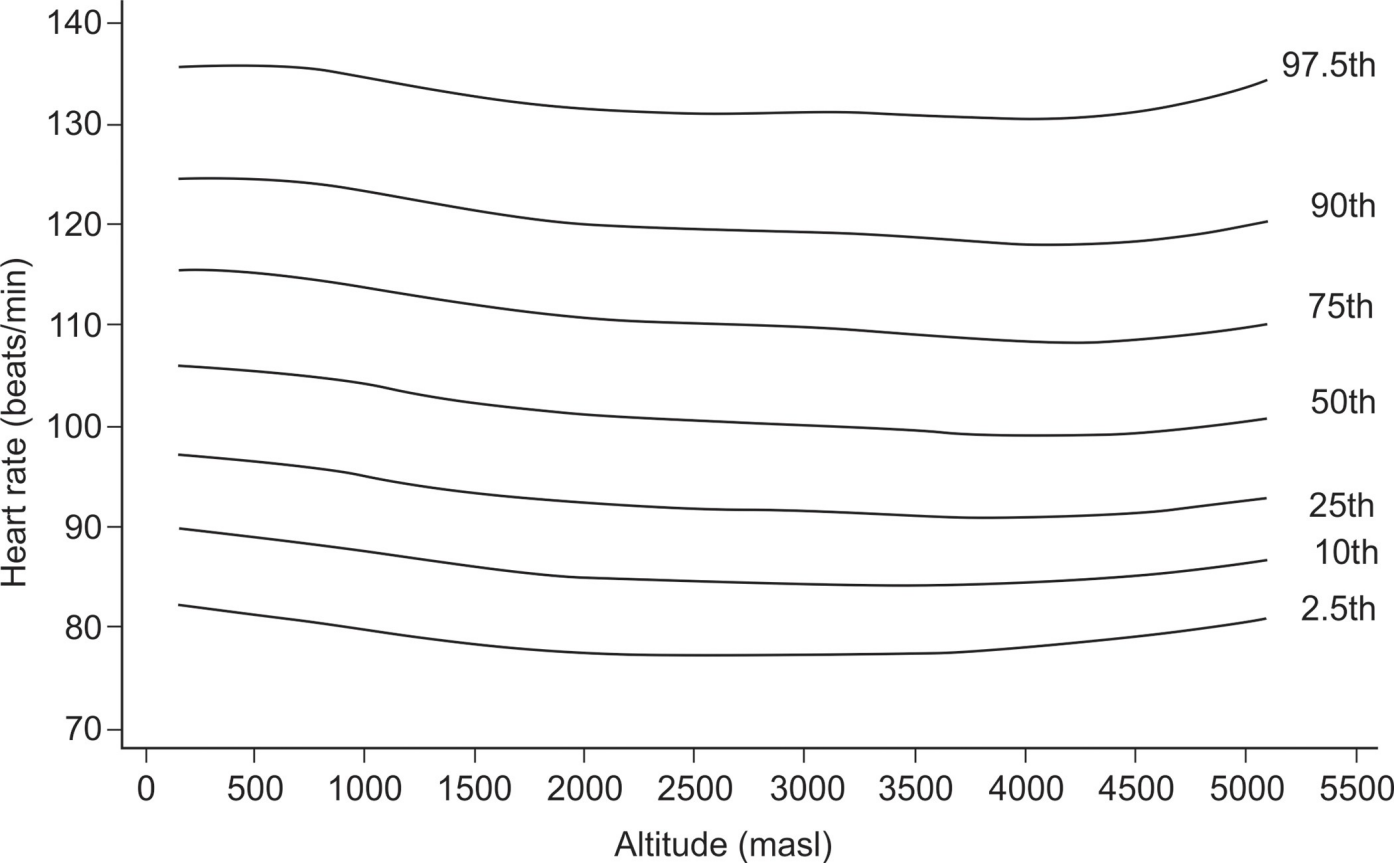
Heart rate at rest according to altitude of residence in Peruvian children of 6–17 years of age.

**Fig 3:**
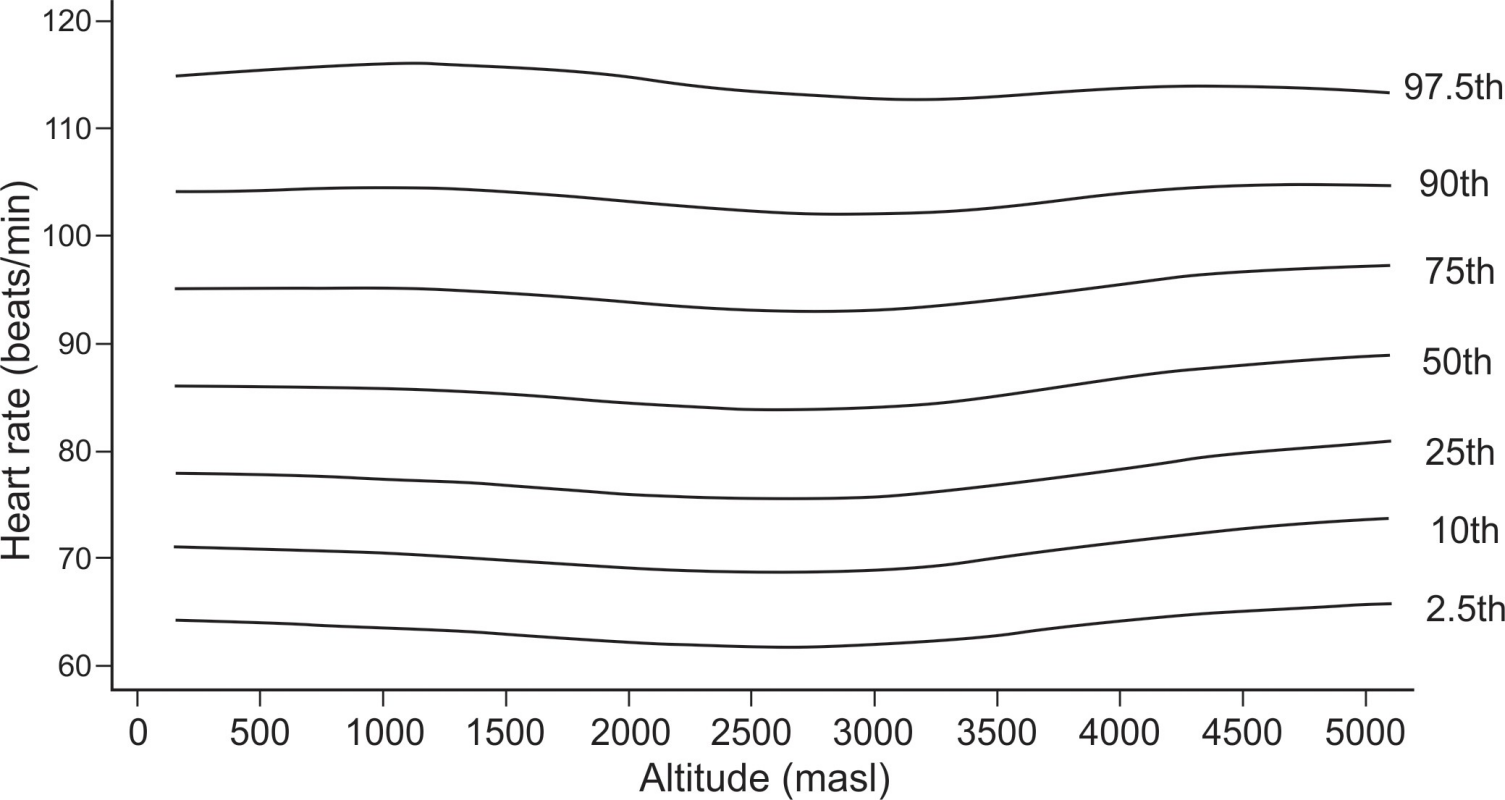
Heart rate at rest according to altitude of residence in Peruvian adults of 18–50 years of age.

**Fig 4:**
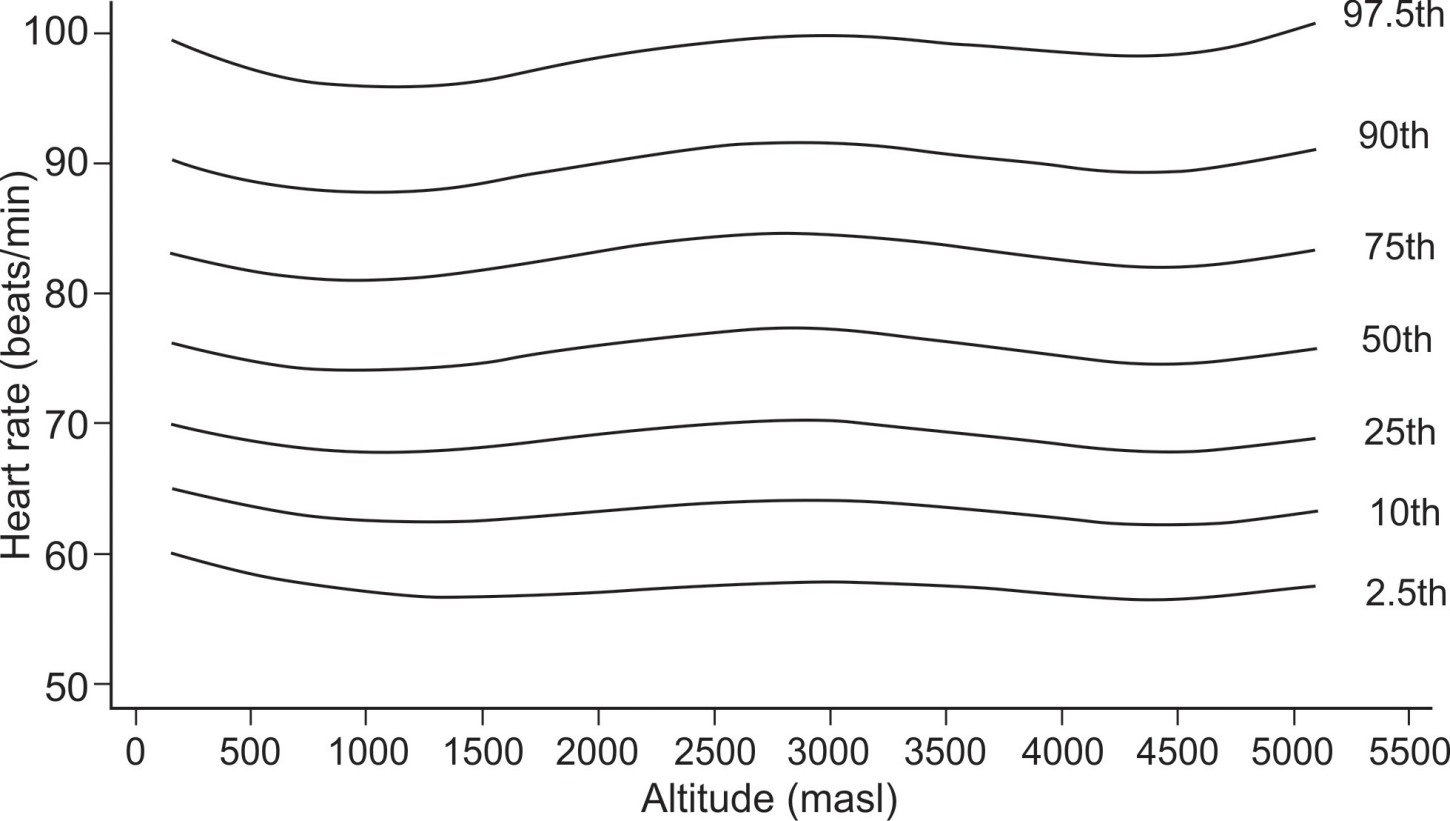
Heart rate at rest according to altitude of residence in Peruvian adults of 51–80 years of age.

**Fig 5:**
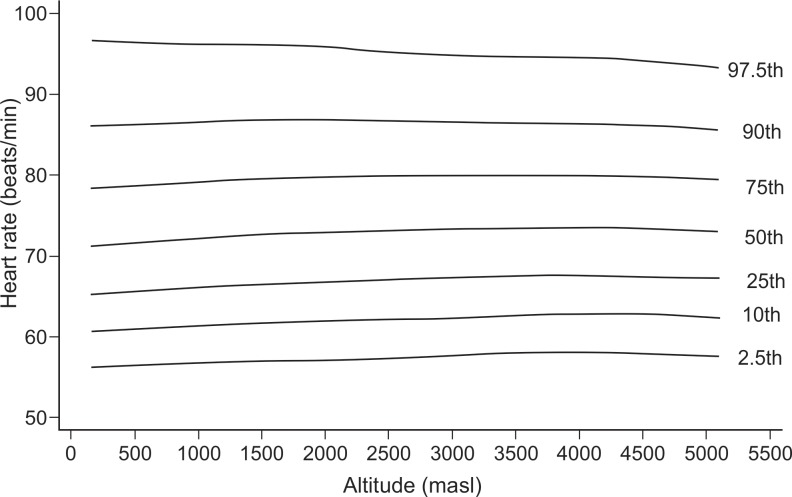
Heart rate at rest according to altitude of residence in Peruvian children and adults.

In exploratory analysis of all age groups in all altitudes combined, the Pearson correlation between HR and altitude showed a coefficient of -0.033 (p-value = 0.009). When we analyzed each age group separately, it was found for the age group of 1–5 years (Fig 1), a coefficient of -0.118 (value p = 0.012), for the group of 6–17 years (Fig 2), it was 0.047 (value p = 0.025), for the group of 18–50 years (Fig 3), it was -0.044 (p value = 0.041) and for the group of 51–80 years (Fig 4), it was 0.042 (p value = 0.256). Despite our finding that 3 out of 4 groups were statistically significant, the variations seemed to be negligible. Fig 5 shows the consolidation of heart rates in the total population evaluated, where you can see curves that vary very little according to residence altitude, especially for values in the range of 60–110 beats per minute.

## Discussion

Our study contained a large sample size which included a broad age range of people from various altitudes. It showed that while the heart rate (HR) had minimal but statistically significant, variations according to the residence altitude in our younger and middle aged patients, the variations were not statistically significant in our elderly population (51–80 years).

In previous studies, it was observed that HR increases with acute hypoxia, [9–15] then returns to their HR as it was at sea level as subjects acclimatize [7, 16, 17]. This can provide an explanation for our results, which were statistically significant due to a large sample, but the differences were negligible.

With our study we analyzed, for the first time, the heart rate values in a broad age range and including all habitable altitudes. Moreover, our study adds information regarding the expected heart rate values at different altitudes using centiles in different age groups, especially children. This is important because current heart rate reference values for children are not based on up to date evidence and recent studies are proposing to describe those values using centile charts [18].The minimal variation of heart rate values in acclimatized subjects is opposed to other physiological measures, which are influenced by altitude of residence, such as oxygen saturation, which decreases as altitude increases [9,19–23].

Our results are important because they can be used in the clinical practice in high altitudes settings. Our study has shown that HR is minimally influenced by the altitude of residence and among different age groups. By displaying the results in centiles charts, it is also easy to use this data for clinical purposes.

Our study has some limitations, one of them being selection bias, which makes it difficult to make an inference about the entire Andean population. Another limitation of our study is that subjects less than 1 year of age and over 80 years of age were not included. Also it was not possible to verify that the subjects were completely healthy since laboratory tests and images were not performed.

With our study, we conclude that, in the age groups of 1–5, 6–17 and 18–50 years of age, the variations in HR were statistically significant due to our large sample size but the variations were found to be negligible. However, in the group of 51–80 years of age, there were no statistically significant differences.
